# Estimation of recurrent atherosclerotic cardiovascular event risk in patients with established cardiovascular disease: the updated SMART2 algorithm

**DOI:** 10.1093/eurheartj/ehac056

**Published:** 2022-02-15

**Authors:** Steven H J Hageman, Ailsa J McKay, Peter Ueda, Laura H Gunn, Tomas Jernberg, Emil Hagström, Deepak L Bhatt, Ph. Gabriel Steg, Kristi Läll, Reedik Mägi, Mari Nordbø Gynnild, Hanne Ellekjær, Ingvild Saltvedt, José Tuñón, Ignacio Mahíllo, Álvaro Aceña, Karol Kaminski, Malgorzata Chlabicz, Emilia Sawicka, Taavi Tillman, John W McEvoy, Emanuele Di Angelantonio, Ian Graham, Dirk De Bacquer, Kausik K Ray, Jannick A N Dorresteijn, Frank L J Visseren

**Affiliations:** 1 Department of Vascular Medicine, University Medical Center Utrecht, PO Box 85500, 3508 GA Utrecht, The Netherlands; 2 Department of Primary Care and Public Health, Imperial College London, London, UK; 3 Clinical Epidemiology Division, Department of Medicine, Karolinska Institutet, Solna, Stockholm, Sweden; 4 Department of Public Health Sciences and School of Data Science, University of North Carolina at Charlotte, Charlotte, NC, USA; 5 Department of Clinical Sciences, Danderyd Hospital, Karolinska Institutet, Stockholm, Sweden; 6 Department of Medical Sciences, Uppsala University, Uppsala Clinical Research Center, Uppsala, Sweden; 7 Brigham and Women's Hospital Heart and Vascular Center, Harvard Medical School, Boston, MA, USA; 8 French Alliance for Cardiovascular Trials, Assistance Publique-Hôpitaux de Paris, Hôpital Bichat, Université de Paris, INSERM Unité, 1148 Paris, France; 9 Estonian Genome Centre, Institute of Genomics, University of Tartu, Tartu, Estonia; 10 Department of Neuromedicine and Movement Science, Faculty of Medicine and Health Science, NTNU—Norwegian University of Science and Technology, Trondheim, Norway; 11 Department of Stroke, Clinic of Medicine, St Olavs Hospital, Trondheim University Hospital, Trondheim, Norway; 12 Department of Geriatrics, Clinic of Medicine, St Olavs Hospital, Trondheim University Hospital, Trondheim, Norway; 13 Department of Cardiology, Fundación Jiménez Díaz, Madrid, Autónoma University, Madrid, Spain; 14 CIBERCV, Madrid, Spain; 15 Department of Epidemiology, Fundación Jiménez Díaz, Madrid, Spain; 16 Department of Population Medicine and Lifestyle Diseases Prevention, Medical University of Bialystok, Białystok, Poland; 17 Department of Invasive Cardiology, Medical University of Bialystok, Białystok, Poland; 18 Department of Cardiology, Medical University of Bialystok, Białystok, Poland; 19 Centre for Non-Communicable Disease, Institute for Global Health, University College London, London, UK; 20 National Institute for Prevention and Cardiovascular Health, Galway, Ireland; 21 Galway Campus, National University of Ireland Galway, Galway, Ireland; 22 Cardiovascular Epidemiology Unit, Department of Public Health and Primary Care, University of Cambridge, Cambridge, UK; 23 School of Medicine, Trinity College Dublin, University of Dublin, Dublin, Ireland; 24 Department of Public Health and Primary Care, Ghent University, Ghent, Belgium

**Keywords:** Risk prediction, Secondary prevention, Established ASCVD, Personalized treatment, Residual risk, Recurrent risk

## Abstract

**Aims:**

The 10-year risk of recurrent atherosclerotic cardiovascular disease (ASCVD) events in patients with established ASCVD can be estimated with the Secondary Manifestations of ARTerial disease (SMART) risk score, and may help refine clinical management. To broaden generalizability across regions, we updated the existing tool (SMART2 risk score) and recalibrated it with regional incidence rates and assessed its performance in external populations.

**Methods and results:**

Individuals with coronary artery disease, cerebrovascular disease, peripheral artery disease, or abdominal aortic aneurysms were included from the Utrecht Cardiovascular Cohort-SMART cohort [*n* = 8355; 1706 ASCVD events during a median follow-up of 8.2 years (interquartile range 4.2–12.5)] to derive a 10-year risk prediction model for recurrent ASCVD events (non-fatal myocardial infarction, non-fatal stroke, or cardiovascular mortality) using a Fine and Gray competing risk-adjusted model. The model was recalibrated to four regions across Europe, and to Asia (excluding Japan), Japan, Australia, North America, and Latin America using contemporary cohort data from each target region. External validation used data from seven cohorts [Clinical Practice Research Datalink, SWEDEHEART, the international REduction of Atherothrombosis for Continued Health (REACH) Registry, Estonian Biobank, Spanish Biomarkers in Acute Coronary Syndrome and Biomarkers in Acute Myocardial Infarction (BACS/BAMI), the Norwegian COgnitive Impairment After STroke, and Bialystok PLUS/Polaspire] and included 369 044 individuals with established ASCVD of whom 62 807 experienced an ASCVD event. *C*-statistics ranged from 0.605 [95% confidence interval (CI) 0.547–0.664] in BACS/BAMI to 0.772 (95% CI 0.659–0.886) in REACH Europe high-risk region. The clinical utility of the model was demonstrated across a range of clinically relevant treatment thresholds for intensified treatment options.

**Conclusion:**

The SMART2 risk score provides an updated, validated tool for the prediction of recurrent ASCVD events in patients with established ASCVD across European and non-European populations. The use of this tool could allow for a more personalized approach to secondary prevention based upon quantitative rather than qualitative estimates of residual risk.

## Structured Graphical Abstract

**Structured Graphical Abstract:**
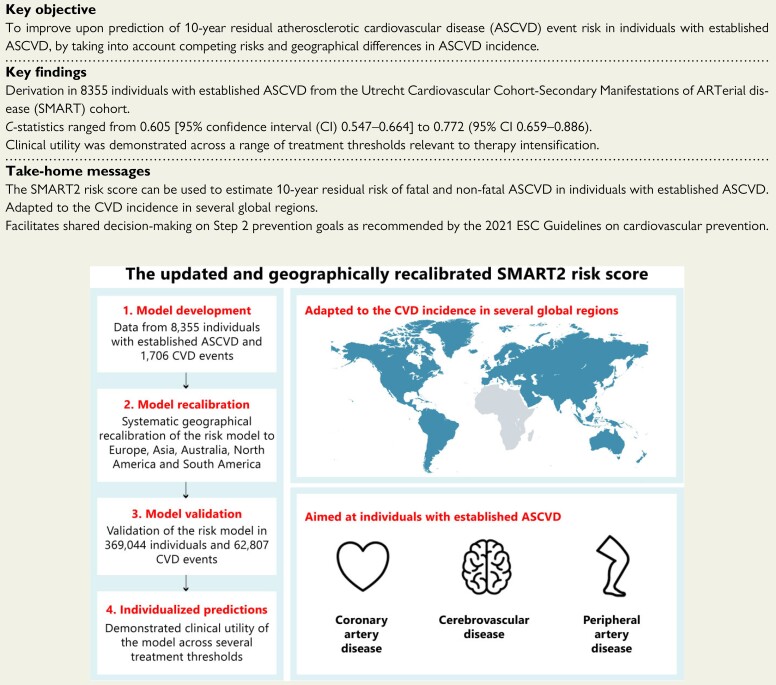
The updated and geographically recalibrated SMART2 risk score.

**See the editorial comment for this article ‘Risk scoring in secondary prevention: a basis for informed clinical decisions in the context of ever-expanding treatments’, by Naveed Sattar and Paul Welsh, https://doi.org/10.1093/eurheartj/ehac125.**

## Introduction

Atherosclerotic cardiovascular diseases (ASCVD), such as coronary heart disease and cerebrovascular disease, are the most common non-communicable diseases globally, and were responsible for an estimated 17.8 million deaths worldwide in 2017.^1^ Clinical guidelines advocate the use of risk prediction models in patients without vascular disease or diabetes, since those at high risk of ASCVD are more likely to benefit from preventive strategies.^2–4^ Clinical guidelines have traditionally advised classification of all patients with established vascular disease as being at ‘very high risk’ for future (recurrent) ASCVD events.^5–7^ This universal approach to allocating risk among secondary prevention patients ignores the fact that the individual level of cardiovascular disease (CVD) risk can vary in these patients^8^ and precludes the option for a more personalized approach to risk factor management in secondary prevention. More intensive treatment options, such as lower treatment targets for blood pressure and LDL-cholesterol, or additional antithrombotic strategies have been proven to further reduce the risk of ASCVD events. However, their implementation has been generally modest, in part reflecting uncertainties about cost benefits from implementing these at scale or uncertainties about individual risk-benefits such as the risk of major bleeding. This makes the identification of patients who may benefit most from more intensive therapy a key issue in clinical practice today.^9,10^ For this reason, more recent European Society of Cardiology (ESC) guidelines now recommend that clinicians consider including information on risk to help inform clinician–patient joint decision-making for secondary prevention treatments.^7,11^

For patients with established ASCVD, the 10-year risk of recurrent ASCVD can be estimated with the previously published Secondary Manifestations of ARTerial disease (SMART) risk score.^12^ The SMART risk score was developed using the Utrecht Cardiovascular Cohort-SMART disease (UCC-SMART)^13^ and externally validated in several trials and routine care populations.^8,14,15^ It was made available via online calculators on the ESC website, the ESC CVD risk prediction app, and U-prevent.com. However, the SMART risk score has several limitations. First, the model was derived using data from participants recruited before 2010 and followed for a median of 4.7 years, and hence may not be directly applicable to predicting 10-year risk in contemporary populations. Second, the model has no parameter to reflect regional differences in CVD incidence, possibly limiting the applicability of the prediction model to the low-risk region where it was developed. Third, the SMART risk score does not take competing risk for non-CVD death into account, which might lead to an overestimation of ASCVD risk in patients at higher risk of competing ‘non-CVD’ death, such as older individuals.^16^ Therefore, we set out to update the SMART risk score by providing derivation (taking competing risk into account), geographic recalibration, and external validation of the new risk score (SMART2) to estimate 10-year residual ASCVD event risk in patients with established ASCVD aged 40–80 years.

## Methods

### Population

Following the previous version of the SMART risk score, the target population for the SMART2 risk score consists of individuals with stable, established ASCVD. The SMART2 risk score was developed using patients with established ASCVD from the UCC-SMART cohort aged 40–80 years. UCC-SMART cohort is a single-centre ongoing prospective cohort study at the University Medical Center Utrecht, The Netherlands.^13^ Patients newly referred to the University Medical Centre Utrecht with established ASCVD, or an increased risk thereof, were included in the period 1996–2019. For the current analysis, we included patients with a history of any type of established ASCVD; which comprised of coronary artery disease (CAD), cerebrovascular disease (CeVD), peripheral artery disease (PAD), and/or abdominal aortic aneurysm (AAA). Coronary artery disease was defined as angina pectoris with documented stenosis, myocardial infarction, or coronary revascularization (coronary bypass surgery or coronary angioplasty); CeVD as a transient ischaemic attack, cerebral infarction, amaurosis fugax or retinal infarction, or a history of carotid surgery; PAD was defined as a symptomatic and documented obstruction of distal arteries of the leg or a history of vascular surgery of the leg (percutaneous transluminal angioplasty, bypass, or amputation); and patients with AAA had a supra- or infrarenal aneurysm of the aorta (distal aortic anteroposterior diameter ≥3 cm, measured at baseline examination with ultrasonography) or a history of AAA surgery. All baseline characteristics were determined using a standardized screening protocol consisting of questionnaires, physical examination, and laboratory testing.

For external validation, patients were included from the Clinical Practice Research Datalink (CPRD) in the UK,^17^ the international REduction of Atherothrombosis for Continued Health (REACH) Registry,^18–20^ the Bialystok PLUS/Polaspire cohort from Poland,^21^ the Estonian Biobank,^22^ Spanish Biomarkers in Acute Coronary Syndrome and Biomarkers in Acute Myocardial Infarction (BACS/BAMI),^23^ the Norwegian COgnitive Impairment After STroke (Nor-COAST) study,^24^ and the SWEDEHEART registry.^25^ Detailed descriptions of the external validation cohorts can be found in the Supplementary material online, *Methods*. Where possible, predictor definitions were the same as in the derivation data. Disease history variables were based on questionnaires (REACH registry, Bialystok PLUS/Polaspire, BACS/BAMI) or linkage to hospital records or primary care (CPRD, Estonian Biobank, Nor-COAST, SWEDEHEART). Endpoints were followed-up by linkage to primary care records, hospital records, or disease/mortality registries (CPRD, Estonian Biobank, Nor-COAST, SWEDEHEART, BACS/BAMI, Bialystok PLUS/Polaspire), or by annual questionnaires (REACH registry).

### Statistical analyses

The SMART2 coefficients were estimated using Fine and Gray competing risk-adjusted subdistribution hazard model.^26^ This model was chosen as it requires no assumptions regarding the shape of the baseline survival function, whereas it can reliably correct for competing risks.^26^ The primary outcome was the occurrence of new ASCVD events, defined as the composite of non-fatal myocardial infarction, non-fatal stroke, and vascular death (see Supplementary material online, *Table S1*). The SMART2 risk score used the same predictors as the original SMART model: baseline age; sex; current smoking; diabetes mellitus; systolic blood pressure (in mmHg); non-HDL-cholesterol (in mmol/L); presence of CAD, CeVD, PAD, or AAA; estimated glomerular filtration rate (eGFR) (mL/min/1.73 m^2^); high-sensitivity C-reactive protein (hsCRP; mg/L); and years since first clinical manifestation of ASCVD (CAD, CeVD, PAD, or AAA). To account for the use of aspirin or equivalent antithrombotic drugs at baseline (including other antiplatelet drugs and oral anticoagulant drugs), the effect of the drugs was added to the model as a fixed predictor^27,28^ (offset term) with a hazard ratio of 0.81.^29,30^ Antithrombotic therapy use was treated as a fixed predictor because it is intended that decisions guided by the risk score may involve use of these drugs (especially the initiation of dual pathway inhibition); as such they could not be included in the model as a regular predictor. Using the same predictors as the original SMART score would require 34 events per parameter with a total of 544 CVD events. The baseline survival was obtained by predicting the cumulative survival from the SMART2 model based on derivation data mean risk factor levels with the predictEventProb function (*pec* package) in R. To check whether the association of continuous predictors with the outcome variable was adequately explained with a log-linear relationship, Akaike information criterions were used to compare log-linear model fits to log transformations, squared transformations, or restricted cubic splines. Based on this, log transformations were used for non-HDL-cholesterol and hsCRP, and squared transformations for years since the first ASCVD diagnosis and eGFR, no predictors showed the best model fit by using restricted cubic splines. Internal validation discrimination and calibration slope were evaluated by 10-fold cross-validation. Handling of missing data is described in the Supplementary material online, *Methods*.

### Regional recalibration

The SMART2 risk score was recalibrated to four risk regions within Europe, which were grouped based on age- and sex-standardized ASCVD mortality rates identical to the grouping used for SCORE2 (see Supplementary material online, *Figure S1*).^31,32^ Details about the risk regions within Europe are shown in the Supplementary material online, *Methods*. The model was recalibrated to four risk regions within Europe by recalibrating the baseline hazard (shifting with a single multiplicative constant per region) of the SMART2 risk score to the data source in the region deemed most representative. First, the expected-observed ratio was calculated in the recalibration data, by dividing the mean predicted risk by the observed cumulative incidence of ASCVD. Then, the baseline hazard was recalibrated by implementing this expected-observed ratio from the target region in the formula for individual risk predictions (see Supplementary material online, *Tables S2* and *S3*). For the low-risk region (CPRD, *n* = 240 443) and the moderate-risk region (SWEDEHEART, *n* = 67 428), large, contemporary data sources were available with minimal selection. In the other regions, the model was recalibrated to local clinical practice by averaging the recalibration factors of the different cohorts in the region (if multiple cohorts available). For the high-risk region, the Estonian Biobank (*n* = 12 986), Bialystok PLUS/Polaspire (*n* = 219), and REACH Europe high-risk region (Hungary, *n* = 836) were used for recalibration; and for the very high-risk region, the REACH Registry (Bulgaria, Russia, Lithuania, Romania, Ukraine; *n* = 4382) was used. Recalibration to regions outside of Europe [North America (*n* = 15 857), Latin America (*n* = 1446), Asia (excluding Japan, *n* = 5396), Japan (*n* = 3745), and Australia (*n* = 1963)] was performed in the REACH Registry.

### External validation

Calibration was assessed visually using predicted vs. observed risk plots—showing octiles of predicted risks plotted against ASCVD cumulative incidences, rather than Kaplan–Meier estimates which may overestimate ASCVD incidence in the presence of competing risks.^16^ Where possible, calibration was assessed at 10 years (CPRD, *n* = 240 443; SWEDEHEART, *n* = 67 428; Estonian Biobank, *n* = 12 986) as this is the intended prediction horizon of the SMART2 model. For external validation cohorts with <10 years of follow-up, model performance was assessed using the duration of the last complete year with ≥80% endpoint registration, which was 2 or 3 years for the REACH subcohorts (*n* = 46 507, Japan, Latin America, and Europe low-risk region 3 years, others 2 years), Nor-COAST (*n* = 497), and Bialystok PLUS/Polaspire (*n* = 219), and 6 years for BACS/BAMI (*n* = 964). For prediction of 2-, 3-, and 6-year risks, the SMART2 predictions were based on the 2-, 3-, and 6-year baseline hazards instead of the 10-year baseline hazard (see Supplementary material online, *Table S2*). Discrimination was assessed as an incident *C*-statistic at 10 years of follow-up if viable, else the same prediction horizon was used as calibration. Discrimination results were adjusted for competing risks and calculated using the R-package *timeROC*. For SWEDEHEART and CPRD, this was not feasible, and a cumulative C-statistic was used adjusted for competing risks. Results from the same region were pooled using random-effects models. The potential clinical value of the SMART2 was evaluated using decision curve analyses. For this, the net benefit of treating all individuals with a predicted SMART2 risk equal or greater than the treatment threshold was evaluated across a range of relevant potential treatment thresholds. The clinical benefit was evaluated at 10 years of follow-up and was corrected for competing risks. The analyses were performed using R-function *stdca*.^33^ The intensive treatment options as stated in ‘Step 2’ of the 2021 ESC CVD prevention guidelines generally have high costs or the risk of adverse events and thus these are specifically not recommended for all individuals with established ASCVD.^7^ For these intensified treatment options, the residual risk thresholds of 20% up until 50% 10-year risk of ASCVD events were regarded as clinically relevant. Clinical benefit was estimated in all external validation cohorts with at least 10-year maximum follow-up duration (CPRD, SWEDEHEART, Estonian Biobank).^34^ Treatment intensification based on predicted residual risk by the SMART2 algorithm was compared with the strategies of treatment intensification in all patients and to performing no treatment intensification. To illustrate the distributions of the predicted risk in the different regions, a simulation was performed using the UCC-SMART data. In this illustration, equal risk factor distributions were assumed in order to make the rates comparable. All analyses were performed with R-statistical programming (version 3.5.2, R Foundation for Statistical Computing, Vienna, Austria).

### Sensitivity analyses

Sensitivity analyses were performed to evaluate several aspects in model derivation. The methodology of these analyses is described in detail in the Supplementary material online, *Methods*—validation of all sensitivity analyses was performed in the European REACH data. First, to evaluate the potential benefit of separate model derivation for men and women, the model was derived separately for both sexes. Second, to evaluate whether the discriminative ability of the model predictors was stable over the different anatomical locations of established ASCVD, the model was derived and recalibrated separately for the different locations of established ASCVD (CAD, CevD, and PAD/AAA separately).

## Results

### Model derivation

In the derivation data, 8355 patients from UCC-SMART with established ASCVD were included. The mean age at baseline was 61 ± 9 years old, and 74% were male. Detailed patient characteristics are presented in *Table 1*. In a median of 8.2 years of follow-up [interquartile range (IQR) 4.2–12.5], 1706 ASCVD events and 978 non-cardiovascular deaths were observed. The SMART2 risk score subdistribution hazard ratios are presented in *Table 2*. There were no or minimal violations of the proportional hazards assumptions as assessed visually based on plotted Schoenfeld residuals. The internal validation C-statistic was 0.696 [95% confidence interval (CI) 0.682–0.708] and the internal calibration slope was 1.002 (95% CI 0.984–1.019).

**Table 1:** Patient characteristics of the model derivation population

	UCC-SMART (*n* = 8355)
Male sex	6198 (74%)
Age (years)	61 ± 9
Current smoker	2504 (30%)
Body mass index (kg/m^2^)	27 ± 4
Systolic blood pressure (mmHg)	139 ± 20
Diabetes mellitus	1467 (18%)
Established coronary artery disease	5215 (62%)
Established peripheral artery disease	1459 (17%)
Established cerebrovascular disease	2424 (29%)
Established abdominal aortic aneurysm	706 (8%)
Total cholesterol (mmol/L)	4.6 (3.9–5.5)
HDL-cholesterol (mmol/L)	1.2 (1.0–1.4)
LDL-cholesterol (mmol/L)	2.7 (2.1–3.5)
Triglycerides (mmol/L)	1.4 (1.0–2.0)
Estimated GFR (mL/min/1.73 m^2^)	77 ± 18
hsCRP (mg/dL)	2.0 (1.0–4.4)
Statin	5764 (69%)
Antiplatelet therapy or anticoagulants	6494 (78%)
Event rate per 1000 person-years^a^	24

All data in *n* (%), mean ± standard deviation, or median (interquartile range).

GFR, glomerular filtration rate (calculated with the Chronic Kidney Disease Epidemiology Collaboration formula); hsCRP, high-sensitivity C-reactive protein.

a Event rate of fatal + non-fatal (myocardial infarction, stroke) events per 1000 person-years.

**Table 2:** Subdistribution hazard ratios of the Secondary Manifestations of ARTerial disease 2 risk score

	Subdistribution hazard ratio (95% CI)
Age^a^	1.61 (1.50–1.73)
Male sex	1.33 (1.18–1.50)
Current smoking	1.41 (1.27–1.58)
Systolic blood pressure (per 10 mmHg)	1.02 (0.99–1.04)
Non-HDL-cholesterol (mmol/L)^b^	1.28 (1.19–1.39)
Established diabetes mellitus	1.37 (1.22–1.54)
Established coronary artery disease	1.34 (1.17–1.55)
Established cerebrovascular disease	1.42 (1.24–1.61)
Established peripheral artery disease	1.25 (1.09–1.43)
Established abdominal aortic aneurysm	1.39 (1.19–1.62)
Years since first ASCVD diagnosis^a^	1.18 (1.15–1.20)
Estimated glomerular filtration ratio^a^	0.87 (0.86–0.88)
hsCRP^b^	1.25 (1.17–1.34)

Subdistribution hazard ratios from Fine and Gray models predicting the risk of total (fatal + non-fatal) ASCVD.

ASCVD, atherosclerotic cardiovascular disease; CI, confidence interval; hsCRP, high-sensitivity C-reactive protein.

a Squared ratios, the subdistribution hazard ratios are presented as 3rd vs. 1st quartile.

b Log-transformed ratios, the subdistribution hazard ratios are presented as 3rd vs. 1st quartile.

### External validation

External validation of risk models involved data from 369 044 individuals with established ASCVD, recruited into seven cohorts in which 62 807 ASCVD events were observed. Of these, 340 637 (92%) were recruited in Europe. Median follow-up times ranged from 1.9 years (IQR 1.8–1.9) for REACH to 6.5 years (IQR 0.7–9.9) for the Estonian Biobank. Detailed patient characteristics of the included patients are presented in *Table 3*.

**Table 3:** Patient characteristics in the external validation populations

	CPRD (*n* = 240 443)	SWEDEHEART (*n* = 67 428)	Nor-COAST (*n* = 497)	Bialystok PLUS/Polaspire (*n* = 219)	BACS/BAMI (*n* = 964)	Est BB (*n* = 12 986)	REACH (EU) (*n* = 18 100)	REACH (non-EU) (*n* = 28 407)
Male sex	149 433 (62%)	50 062 (74%)	306 (62%)	167 (76%)	735 (76%)	5350 (41%)	13 046 (72%)	19 028 (67%)
Age (years)	66 ± 9	62 ± 9	68 ± 9.5	65 ± 8	61 ± 12	63 ± 10	65 ± 9	67 ± 9
Current smoker	46 790 (19%)	8681 (13%)	62 (12%)	41 (19%)	134 (14%)	2070 (16%)	3307 (18%)	4020 (14%)
Body mass index (kg/m^2^)	28 ± 5	28 ± 5	27 ± 4	30 ± 5	29 ± 4	29 ± 4	28 ± 4	28 ± 6
Systolic blood pressure (mmHg)	139 ± 20	133 ± 20	139 ± 19	134 ± 20	135 ± 21	135 ± 18	141 ± 20	134 ± 19
Diabetes mellitus	38 346 (16%)	17 690 (26%)	97 (20%)	64 (29%)	232 (24%)	2321 (18%)	5749 (32%)	11 955 (42%)
Coronary artery disease	152 279 (63%)	67 428 (100%)	83 (17%)	219 (100%)	964 (100%)	10 668 (82%)	12 871 (71%)	20 856 (73%)
Peripheral artery disease	31 803 (13%)	1142 (2%)	38 (8%)	21 (10%)	36 (4%)	1709 (14%)	3681 (20%)	3149 (11%)
Cerebrovascular disease	71 853 (30%)	3614 (5%)	497 (100%)	17 (8%)	27 (3%)	3314 (25%)	5951 (33%)	9385 (33%)
Abdominal aortic aneurysm	6977 (3%)	474 (1%)	22 (4%)	0 (0%)	4 (0%)	109 (0%)	550 (3%)	930 (3%)
Years since first CVD diagnosis	0.5 (0.5–4.7)	0.2 (0.1–0.2)		2.0 (1.0–5.0)	0.6 (0.5–0.9)	4.8 (2.0–9.5)		
Total cholesterol (mmol/L)	4.7 (4.0–5.6)	4.0 (3.4–4.7)	3.9 (3.4–4.4)	4.0 (3.3–4.7)	3.9 (3.4–4.4)	5.5 (4.8–6.4)	5.2 (4.4–6.0)	4.7 (4.0–5.4)
HDL-cholesterol (mmol/L)	1.3 (1.1–1.6)	1.1 (0.9–1.4)	1.4 (1.1–1.7)	1.2 (1.1–1.6)	1.0 (0.9–1.2)	1.3 (1.0–1.7)		
LDL-cholesterol (mmol/L)		2.1 (1.6–2.7)	2.0 (1.6–2.5)	2.2 (1.7–2.7)	2.0 (1.7–2.4)	3.1 (2.4–3.7)		
Triglycerides (mmol/L)		1.2 (1.0.9–1.7)	1.2 (0.9–1.7)	1.2 (0.8–1.7)	2.6 (2.0–3.6)		1.6 (1.1–2.2)	1.5 (1.1–2.2)
Estimated GFR (mL/min/1.73 m^2^)	67 ± 18	83 ± 19	78 ± 18	88 ± 21	78 ± 18	81 ± 19	73 ± 21	71 ± 23
hsCRP (mg/dL)		5.0 (2.0–9.0)	1.8 (0.8–3.6)	1.1 (0.5–2.5)	1.8 (0.8–3.6)	2.3 (1.1–5.0)		
Statin		65 075 (95%)	434 (87%)	231 (90%)	914 (94.8%)	4181 (32%)	12 483 (69%)	20 156 (71%)
Antiplatelet therapy or anticoagulants	179 129 (75%)	67 049 (99%)	448 (98%)	211 (96%)	902 (94%)		12 646 (70%)	20 831 (73%)
Follow-up (years)	5.3 (2.2–9.6)	4.0 (1.8–6.9)	2.2 (1.8–2.7)	2.9 (2.5–3.5)	4.7 (2.3–6.7)	6.5 (0.7–9.9)	1.9 (1.8–1.9)	1.9 (1.5–1.9)
CVD events	44 985 (19%)	9270 (13%)	54 (11%)	20 (9%)	130 (13%)	3489 (39%)	2201 (12%)	2658 (9%)
Event rate per 1000 person-years^a^	34	31	51	33	30	48	63	50

All data in *n* (%), mean ± standard deviation, or median (interquartile range). GFR, glomerular filtration rate (calculated with the Chronic Kidney Disease Epidemiology Collaboration formula); CVD, cardiovascular disease; hsCRP, high-sensitivity C-reactive protein.

a Event rate of fatal + non-fatal (myocardial infarction, stroke) events per 1000 person-years.

**Figure 1:**
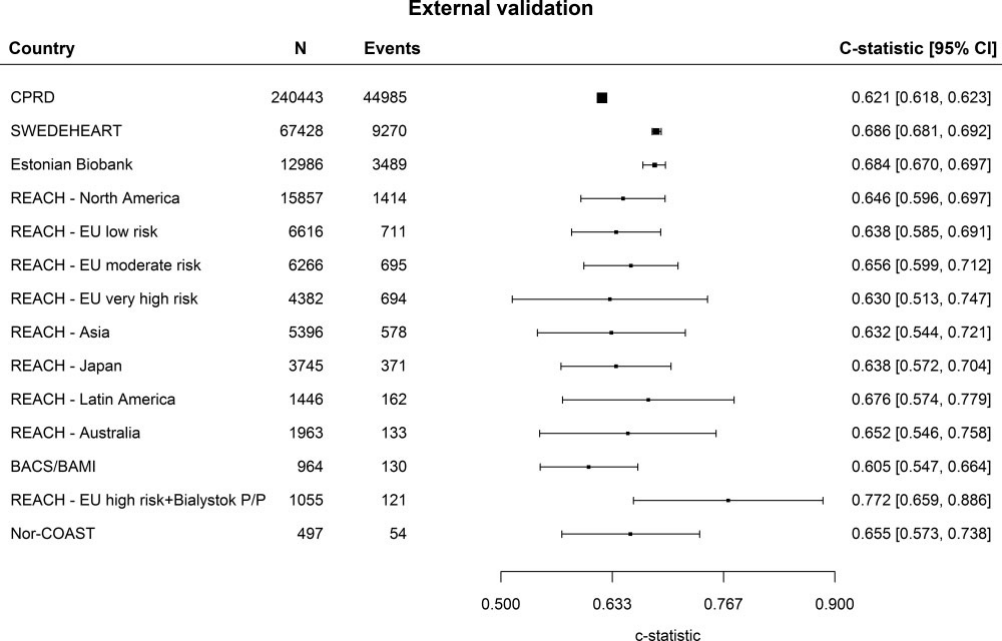
Discrimination in the external validation cohorts. Discrimination in all external validation cohorts based on Harrell’s *C*-statistic.

C-statistics ranged from 0.605 (95% CI 0.547–0.664) in BACS/BAMI, to 0.772 (95% CI 0.659–0.886) in REACH Europe high-risk region (*Figure 1*). Most heterogeneity in discrimination results was found in data from Western Europe. The prediction interval of the C-statistics was 0.646 (95% CI 0.581–0.710) in Western Europe, 0.685 (95% CI 0.670–0.699) in Eastern Europe, and 0.646 (95% CI 0.613–0.679) in the regions outside of Europe (see Supplementary material online, *Figure S2*).

**Figure 2:**
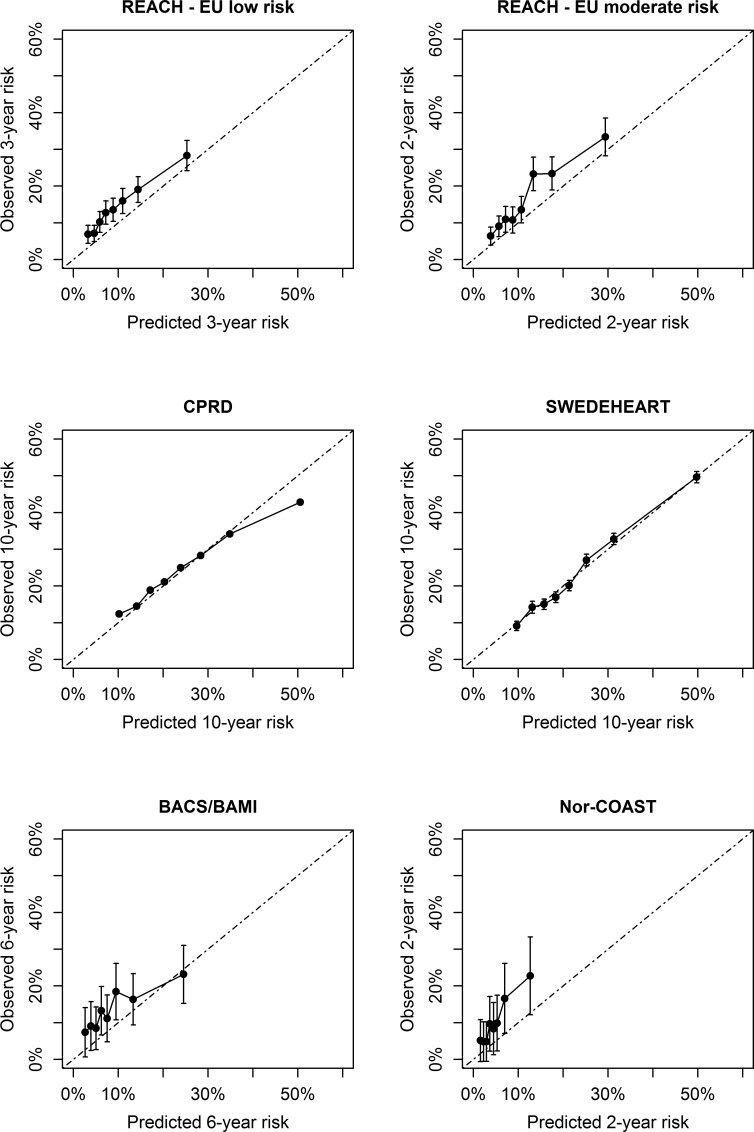
Calibration in external validation cohorts from Western Europe.

**Figure 3:**
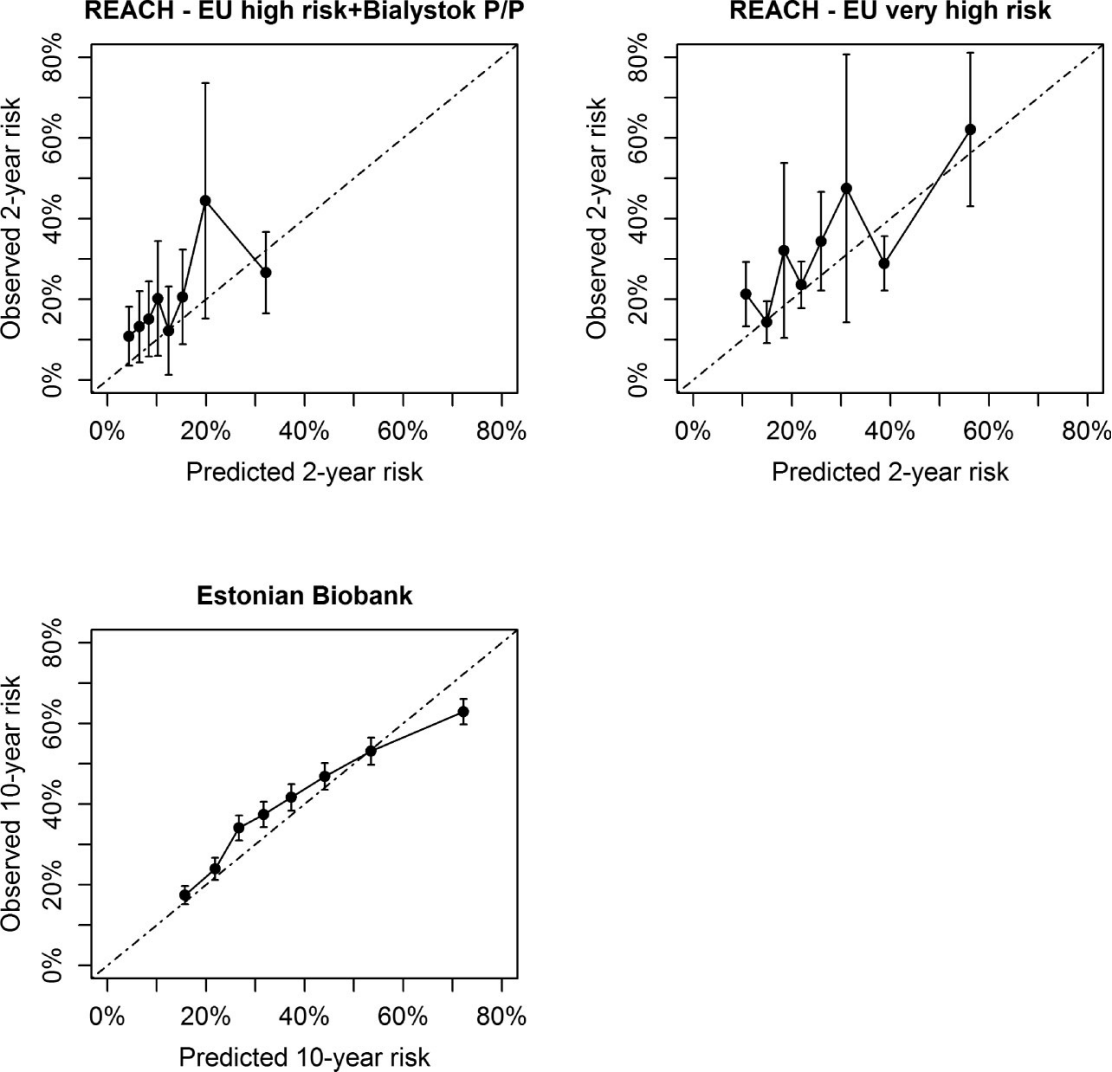
Calibration in external validation cohorts from Eastern Europe.

**Figure 4:**
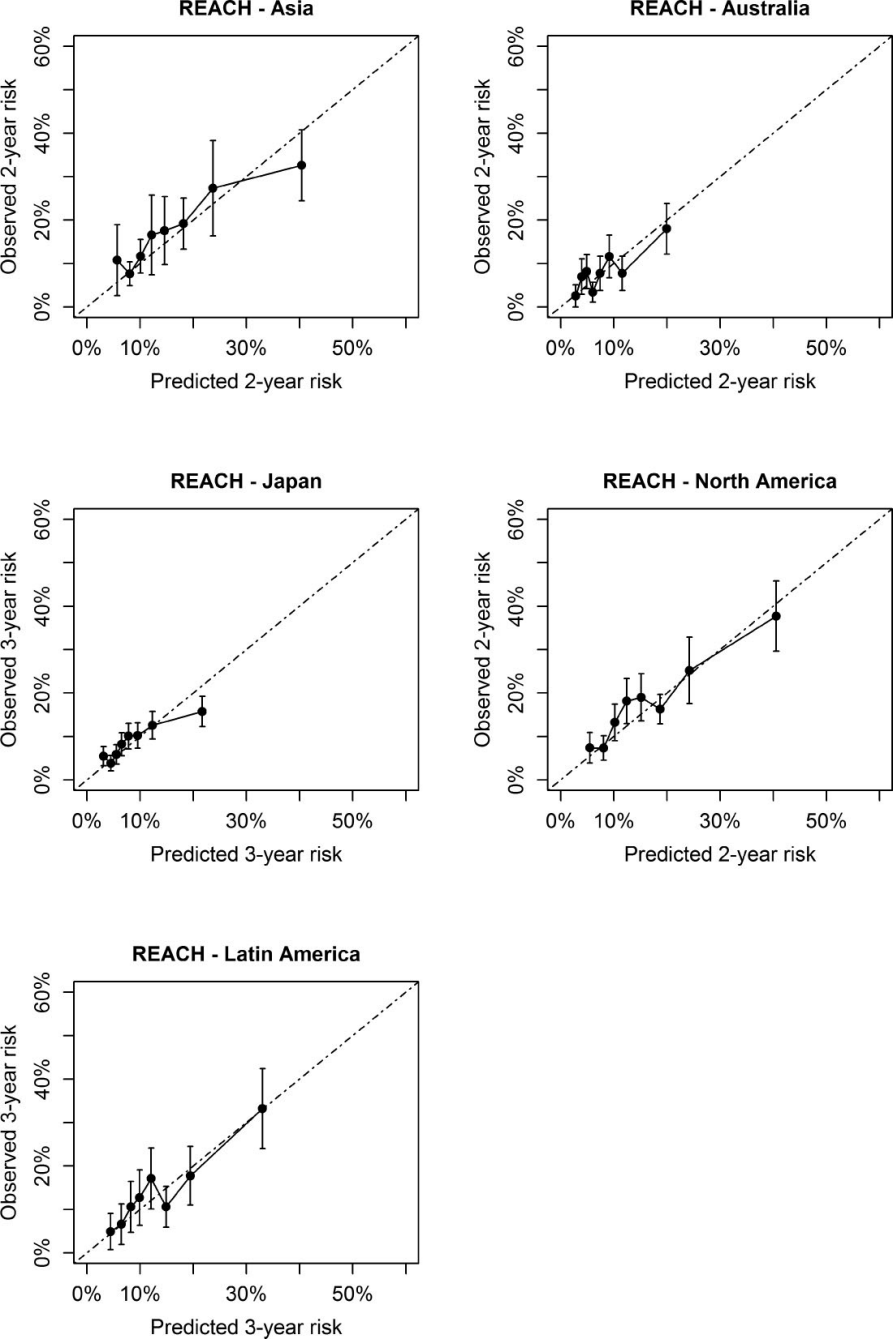
Calibration in non-European external validation cohorts.

Prior to recalibration, there was a systematic underestimation of ASCVD risk in most external validation cohorts (see Supplementary material online, *Figures S3–S5*). After recalibration, in CPRD (low risk), SWEDEHEART (moderate risk), REACH high-risk region and very high-risk region, and the Estonian Biobank (high risk), there were no over- or underestimations in the relevant risk categories (*Figures 2* and *3*). In REACH Europe low and moderate risk regions, Nor-COAST (moderate risk), and BACS/BAMI (low risk), an underestimation of predicted risks was observed. In all regions outside of Europe, no over- or underestimation was observed of the predicted risks (*Figure 4*). All model parameters used for individual risk prediction or recalibration are shown in Supplementary material online, *Table S2*.

### Clinical utility

Results from the decision curve analyses are shown in Supplementary material online, *Figure S6*. Clinical utility of treatment intensification based on SMART2 was superior in all three evaluated cohorts to the other evaluated strategies for scenarios where the intervention was indicated for individuals whose risk of recurrence was 20% or greater—up until scenarios where the intervention was indicated for individuals whose risk of recurrence was 50% or greater. Scenarios evaluating treatment thresholds of <15% 10-year ASCVD risk, relevant for interventions with very low costs and almost no harm, showed similar clinical utility of treating all individuals and personalized treatment based on SMART2. For the thresholds above 50%, mostly relevant for interventions with severe disadvantages, clinical utility of SMART2 was similar to performing no additional treatment intensification in CPRD and SWEDEHEART, and superior to no additional treatment intensification in the Estonian Biobank until the treatment threshold of 60%. The expected proportion of individuals which would be treated using a 20 or 40% treatment threshold in every European risk region is shown in Supplementary material online, *Figure S7*.

### Sensitivity analyses

Sensitivity analyses of REACH data from Western Europe (*n* = 12 882) demonstrated that sex-specific and location-specific model derivations and recalibrations did not improve discriminative model performance (see Supplementary material online, *Table S4*).

## Discussion

The current report describes the development, recalibration, and external validation of the SMART2 risk score for the prediction of recurrent ASCVD in patients with established ASCVD. The model was recalibrated to four risk regions within Europe and for regions outside Europe, and external validation was performed in all these regions (*Structured Graphical Abstract*). The clinical utility of the SMART2 model was demonstrated across a range of clinically relevant treatment thresholds in several of these regions.

The SMART2 risk score includes features that confer advantages compared with the original SMART risk score and other existing tools, such as the SMART-REACH model or the recently published EUROASPIRE risk calculator.^14,35^ First, the SMART2 risk score is underpinned by large, and extensive datasets from multiple countries, used for model derivation, recalibration, and validation. Models were derived and externally validated using cohorts and registries with long-term follow-up, during which large numbers of hard vascular endpoints were observed—in total 64 513 CVD events in 377 399 individuals with established ASCVD. The cohorts represent different clinical manifestations of ASCVD, including diseases of the coronary, cerebral, and peripheral circulation. This provides greater generalizability of the derived model and validation results and therefore more likely reflects unmet clinical needs particularly in the generalist settings. As both the model derivation and validation populations of the current study included individuals with polyvascular disease (i.e. those with established ASCVD at multiple locations), the SMART2 risk score can be applied to this high-risk population as well.

Moreover, an important strength of the SMART2 risk score is the use of easy-to-measure variables, which are for the most part routinely measured as part of routine clinical practice. This makes it more likely that SMART2 risk tool is clinically applicable to busy, routine practice. Where variables have not been collected in clinical practice, like hsCRP for example, automated imputation of these individual risk factor values is possible by using mean values of the derivation dataset. This allows estimates of risk to be generated with acceptable prediction metrics,^15,36^ a user-friendly function which is already incorporated in online calculators like the ESC CVD risk prediction app or http://U-prevent.com, and the U-Prevent smartphone app. Although the concept of estimating 10-year risk in secondary prevention, with which to guide treatment intensification is relatively new as a concept and has not been formally tested in clinical outcome trials, the increasingly expensive therapeutic armamentarium that is available to treat secondary prevention patients, and the finite resources with which to treat them, makes the use of such risk estimation tools to personalize treatment decisions more attractive. Furthermore, clinicians already use a similar approach in primary prevention with 10-year estimates of CVD risk in order to guide first-line therapies. Therefore, using the same approach in secondary prevention and variables that clinicians already measure makes utilization more likely.

Third, possibly the most important update of the SMART2 risk score is that the risk model is geographically recalibrated to multiple different risk regions, both within and outside of Europe. This provides further assurance that the risk model is reliable in local clinical practice settings across multiple geographical locations. On average, the original SMART model performed adequately in contemporary Western European populations, and a systematic underestimation of predicted risk was seen in Eastern European countries,^15,35^ similar to what has been observed in primary prevention settings with SCORE. In the current SMART2 update, however, the model was recalibrated to four European risk regions and to North America, Latin America, Asia (excluding Japan), Japan, and Australia. Results from the current study show external validation in terms of discrimination and calibration in all these regions. In all regions which had a cohort available with a least 10 years of follow-up (Europe’s low, moderate, and high-risk region), clinical utility of the SMART2 risk score was demonstrated across a range of clinically relevant treatment thresholds, indicating the usefulness in clinical practice.

Fourth, the SMART2 risk score accounts for the impact of competing risks—which confers an important advantage in comparison to the original SMART score or the EUROASPIRE risk calculator. As the intended age-range of the SMART2 risk score reaches 80 years, not accounting for competing risks could greatly overestimate predicted risks and treatment effects, especially in older individuals.^16^ Treatment initiation based on overestimated risks may lead to overly optimistic estimates of the individual effect of preventive treatment options.^37^ Importantly, competing risk-adjusted risk estimates better reflect the way that risk is generally interpreted in clinical practice: the probability of having an ASCVD event in the next 10 years. In contrast, unadjusted risk prediction (i.e. those originating from Cox proportional hazard models) should be explained as the probability of having an ASCVD event in the hypothetical situation of immortality to other causes of death during the next 10 years.^37,38^

The SMART2 risk algorithm could help resolve clinical uncertainties, and potentially improve clinical practice and treatment inertia by better quantifying risk, thus identifying those patients who may benefit most from additional preventive strategies. Traditionally, all patients with established ASCVD are classified as very high risk, and the same preventive measures are advised for all of them.^2^ However, even after treating risk factor levels to evidence-based secondary prevention targets, significant residual risk may remain and there is large individual variation of residual risk in this population.^8^ The SMART2 risk score may help to identify those at the highest residual risk who are likely to benefit most from treatment intensification. Further intensification of preventive interventions has the advantage of lowering ASCVD risk, but may have disadvantages like polypharmacy, increased costs, and potential harms, like bleeding risks in the case of antithrombotic therapies. By combining 10-year risk predictions with intensified treatment effects from lipid lowering, blood pressure, or anticoagulant therapy, treatment effects can be estimated.^3,39^ These treatment effects can be used, together with treatment harms and preferences of both patient and health care provider, to inform the shared decision-making process. Current guidelines suggest to consider intensifying preventive treatment based on residual 10-year risk, although no specific treatment thresholds are recommended.^2,7,11^ If future guidelines were to include treatment thresholds to guide residual risk reduction, a contemporary well-calibrated model that is generalizable is required. The SMART2 tool provides such a solution, and was shown to provide clinical utility in those thresholds relevant to further therapy intensification.

The potential limitations of our study merit consideration. First, the SMART2 risk model was derived using data from only a low-risk country. Ideally, the derivation of the risk model would have involved representative prospective cohort data from all target regions, including high-risk regions like Eastern Europe, but this was practically not possible as the different datasets were at different geographical locations and could not be combined into one dataset. However, the effects of predictors on the risk of ASCVD events seemed to be stable across geographical regions,^40,41^ and Eastern European discrimination results were comparable to low-risk regions, indicating that the relative effects of the risk predictors were transferable to other risk regions. As the baseline risk of ASCVD events is different across geographical regions, large contemporary datasets from all target regions were used to recalibrate the model intercept to these regions. There may still be a certain extent of variation in CVD incidence within the risk regions used for recalibration. Further recalibration of the SMART2 risk score to more subregions could be a topic for future research. In addition, the data sources that were used for recalibration to every risk region reflect current incidence rates and treatment patterns. Changing cardiovascular incidence rates and treatment patterns, including changes in antithrombotic treatment, may warrant updates and repeated validation in the future.

Moreover, the model could not be validated on the intended 10-year prediction horizon in all risk regions as this data was only available in Europe’s low, moderate, and high-risk regions. In the other risk regions, a shorter prediction horizon was used to validate the SMART2 risk score. Therefore, the SMART2 risk score may benefit from further long-term validation in these regions. Reassuringly, however, the relative effect of common risk factors on the risk of CVD events is generally stable over time^40^ and the validation results in the cohorts with available 10-year follow-up were adequate. In addition, the cohorts in which 10-year validations were viable were very large in comparison to those validated at short prediction horizons.

Another potential limitation is the use of cohort data in several stages of the analysis. Cohorts often have a healthy participant bias and even within risk regions, there is always some inter-cohort variation in risk factor levels and disease incidence. These differences in incidence rates are not explainable by risk factor levels alone nor do they necessarily reflect biological differences in disease risk. Often, these differences can be explained by differences in patient selection, arising from varying inclusion criteria or methods or by participation rates. In the low-risk region, for example, the UCC-SMART cohort represents an outpatient clinic patient population of individuals with stable established ASCVD. Atherosclerotic cardiovascular disease incidence in UCC-SMART is lower than in Nor-COAST and BACS/BAMI, which are from the same risk region but rather included patients consecutively after recently experiencing stroke or coronary events, leading to higher risk populations. These differences likely explain the underestimation of predicted risk in those cohorts as found in the current study. The SMART2 risk score is intended to inform shared decision-making in patients with established ASCVD, which is often performed in outpatient clinics. Therefore, the model was recalibrated to all risk regions with cohort data resembling outpatient clinic populations where possible.

In conclusion, the derivation, recalibration, and external validation of the SMART2 risk score were shown for the prediction of recurrent ASCVD among patients with established ASCVD. The model was improved by the use of large and contemporary data, recalibration across various regions, and adjustment for competing risks. The use of this tool could allow for a more personalized approach to secondary prevention based upon quantitative rather than qualitative estimates of residual risk.

## Supplementary Material

ehac056_Supplementary_Data
