# Supplementary material for: Estimation of recurrent atherosclerotic cardiovascular event risk in patients with established cardiovascular disease: the updated SMART2 algorithm
Source: Eur Heart J. 2022 Feb 15;43(18):1715–27. doi: 10.1093/eurheartj/ehac056 (PMC9312860; doi:10.1093/eurheartj/ehac056)
Supplement: ehac056_Supplementary_Data [file ehac056_supplementary_data.zip › SMART2_supplement_20211230.docx]

# Extending the scope of a secondary prevention risk tool across Europe and beyond: the updated and geographically recalibrated SMART2 algorithm

Online Supplement

# Supplementary Methods

## External validation cohorts

In the international, prospective REACH Registry, participants were enrolled between 2003 and 2004 from physician outpatient practices in several regions, including Western and Eastern Europe. Participants were followed for a maximum of 4 years for the occurrence of CVD events and mortality. The REACH Registry was used before to assess geographical differences in the risk of recurrent cardiovascular disease.^1^ For European regions, the countries were reclassified to the risk regions as used for the SCORE2 project based on standardized CVD mortality rates. Asian and Middle Eastern REACH regions were merged as the number of included Middle Eastern individuals was low and the rates for Asia and the Middle East were very comparable.

Bialystok PLUS/Polaspire included patients in 2016–2018 who were previously hospitalized for acute coronary event or elective percutaneous revascularisation procedure and were followed for a median time of 3 years. Patients were included in the study 12-18 months after the coronary event. Follow-up was performed through return visits at 1 and 3 years after baseline or by linkage to national mortality registers.

The Estonian Biobank is a population-based study from the Estonian Genome Center at the University of Tartu. From this Biobank, all patients were included with established ASCVD prior to inclusion. All biobank participants have signed a broad informed consent form and the study was carried out under ethical approval 1.1-12/624 from the Estonian Committee on Bioethics and Human Research (Estonian Ministry of Social Affairs) and data release N05 from the EstBB. Data analyzes in Estonian dataset were carried out in part in the High-Performance Computing Center of University of Tartu.

In BACS/BAMI, patients were included when admitted at four hospitals in the area of Madrid with either non-ST elevation acute coronary syndrome or ST elevation myocardial infarction between 2006 and 2010. Follow-up for the current study started at this first follow-up visit, 6-12 months after inclusion events.

SWEDEHEART is a large Swedish nationwide myocardial infarction quality registry including all patients treated at coronary care units in Sweden, as well as data on all patients undergoing coronary revascularization (angiography/angioplasty and coronary bypass grafting). Follow-up information was obtained by linkage to national registries. For the current study, all individuals with myocardial were included who had their follow-up visit between 31/01/2005 and 31/12/2016. Risk factor measurements were used from the follow-up visit 6-10 weeks after this event – follow-up started at this point.

Nor-COAST is a multicentre (5 centres, all in Norway), prospective, cohort study, consecutively including patients with acute stroke between 2015 and 2017. Patients have follow-up visits at 3 and 18 months, and at 3 years. Patients with ischemic stroke were included from 3 months post-stroke – at which point follow-up started for the current study. Follow-up information was obtained by linkage to national registries for cardiovascular disease and mortality.

The Clinical Practice Research Datalink (CPRD) GOLD database is a UK repository containing longitudinal individual primary care patient data collected from 1987 onwards. Over time, 966 primary care centres have contributed data for >20 million patients, with 407 practices actively contributing data for >3 million patients currently. The age, sex and ethnicity distributions of the patient sample broadly reflect those of the general UK population, and linked Hospital Episode Statistics hospital admissions data from English hospitals and Office for National Statistics mortality data, are available. The primary care data are collected during routine general practice activities. The bulk of UK cardiovascular disease prevention work is undertaken in primary care, and (as for other common medical conditions) general practitioners are incentivised to use standard coding procedures to record this activity. The database has previously been used in both derivation and validation of cardiovascular risk prediction tools. For the current study, individuals were eligible to enter the cohort from January 1^st^ 2000 onwards, once they had been registered with the relevant general practice for at least one year (to allow routine reporting to be established), were aged >40 (and <80) years, and were at least six months post their first record of an ASCVD diagnosis.

## Recalibration regions

European regions were grouped on the most recently available (assessed July 2020) age- and sex-standardized overall cardiovascular mortality rates per 100,000 (ICD chapters 9, I00-I99, Supplementary Figure 1)^2^ as follows: low risk (≤100 CVD deaths per 100,000), moderate risk (>100-150 CVD deaths per 100,000), high risk (>150-300 CVD deaths per 100,000), and very high risk (>300 CVD deaths per 100,000). These rates were obtained from the WHO CVD mortality database^2^ to which they were provided by all individual countries.

## Missing data

Because complete case analysis may lead to loss of statistical power and possible bias,^3^ values of the following variables in the derivation data were imputed by single regression imputation using predictive mean matching: smoking status (n=32, 0.4%), creatinine (n=31, 0.3%), hsCRP (n=250, 3.2%), SBP (n=18, 0.2%), HDL-c (n=80, 1.0%), and total cholesterol (n=34, 0.4%).

Different approaches were used for sporadically and systematically missing data. Ideally, systematically missing variables were handled using multilevel multiple imputation with fully conditional specification via the mitml-impute package in R (5 imputed datasets). However, as this required the data being transferred to be combined with the other datasets, this was only possible for the REACH Registry (HDL-c, hsCRP, years since first CVD diagnosis). For systematically missing data in other cohorts (Nor-COAST: AAA) the mean of this variable in the derivation data was used, or with systematically missing aspirin treatment data (Estonian Biobank, CPRD) it was assumed all individuals used aspirin or equivalent treatment. Whereas this approach may lead to biased results in studies assessing variable associations, the effect on goodness-of-fit is limited (assuming roughly similar prevalence of systematic missing risk factors in derivation and validation data), and the approach should lead to a conservative estimate of the C-statistic (as not all model parameters are available for risk stratification, discrimination decreases). In cohorts with only sporadically missing data, these were imputed as in the derivation process using single imputation based on predictive mean matching (R-package aregImpute).

## Sensitivity analyses

Sensitivity analyses were performed to evaluate several aspects in model derivation. First, to evaluate the potential benefit of separate model derivation for men and women, the whole model derivation process was repeated separately for both sexes. The model was then recalibrated separately for both sexes in the REACH Registry Western Europe (all countries in the low or moderate risk region) similar to the recalibration methodology used for the main model. Model performance was assessed in terms of discrimination, both separately for both sexes and using the complete population.

The second sensitivity analyses were similar to the first, but this time derivation and recalibration was performed separate for the different locations of established ASCVD (CAD, CevD, and PAD/AAA separately). Individuals with polyvascular disease contributed to the derivation or recalibration of multiple models (i.e. a subject with both CAD and PAD in UCC-SMART contributed to both the derivation of the CAD-specific and the PAD/AAA-specific model). Individual risk predictions for those with polyvascular disease were calculated by taking the mean of the disease-specific predicted risks (so this individual with CAD and PAD would have two predicted risks: one from the CAD model and one from the PAD/AAA model, the final individual predicted risk is the mean of those two). Model performance was assessed in terms of discrimination, both separately for ASCVD locations and in the complete population.

# References

1. Abtan J, Bhatt DL, Elbez Y, et al. Geographic variation and risk factors for systemic and limb ischemic events in patients with symptomatic peripheral artery disease: Insights from the <scp>REACH</scp> Registry. *Clin Cardiol*. 2017;40(9):710-718. doi:10.1002/clc.22721

2. World Health Organization. WHO Mortality Database. Accessed May 7, 2020. https://apps.who.int/healthinfo/statistics/mortality/whodpms/

3. Donders ART, van der Heijden GJMG, Stijnen T, Moons KGM. Review: A gentle introduction to imputation of missing values. *J Clin Epidemiol*. 2006;59(10):1087-1091. doi:10.1016/j.jclinepi.2006.01.014

# Supplementary Figure 1: European risk regions – similar to SCORE2 risk regions


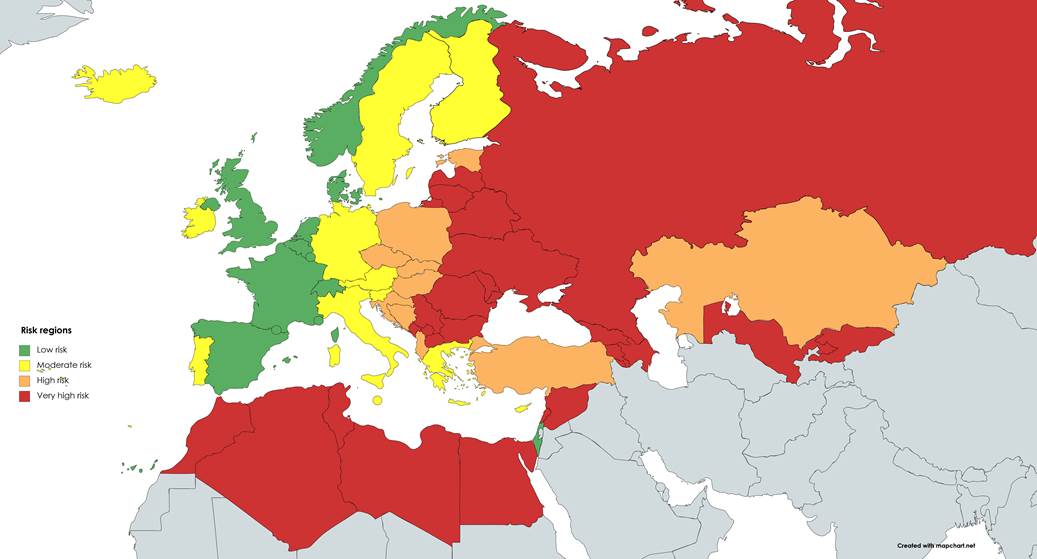


Risk regions based on most recently available age- and sex-standardized overall cardiovascular disease (CVD) mortality rates per 100,000: low risk (≤100 CVD deaths per 100,000), moderate risk (100 to <150 CVD deaths per 100,000), high risk (150 to <300 CVD deaths per 100,000), and very high risk ($\geq$300 CVD deaths per 100,000). Estimates are obtained from the WHO cause specific mortality database (2020).^2^

# **Supplementary Figure 2**: Pooled discrimination results per region


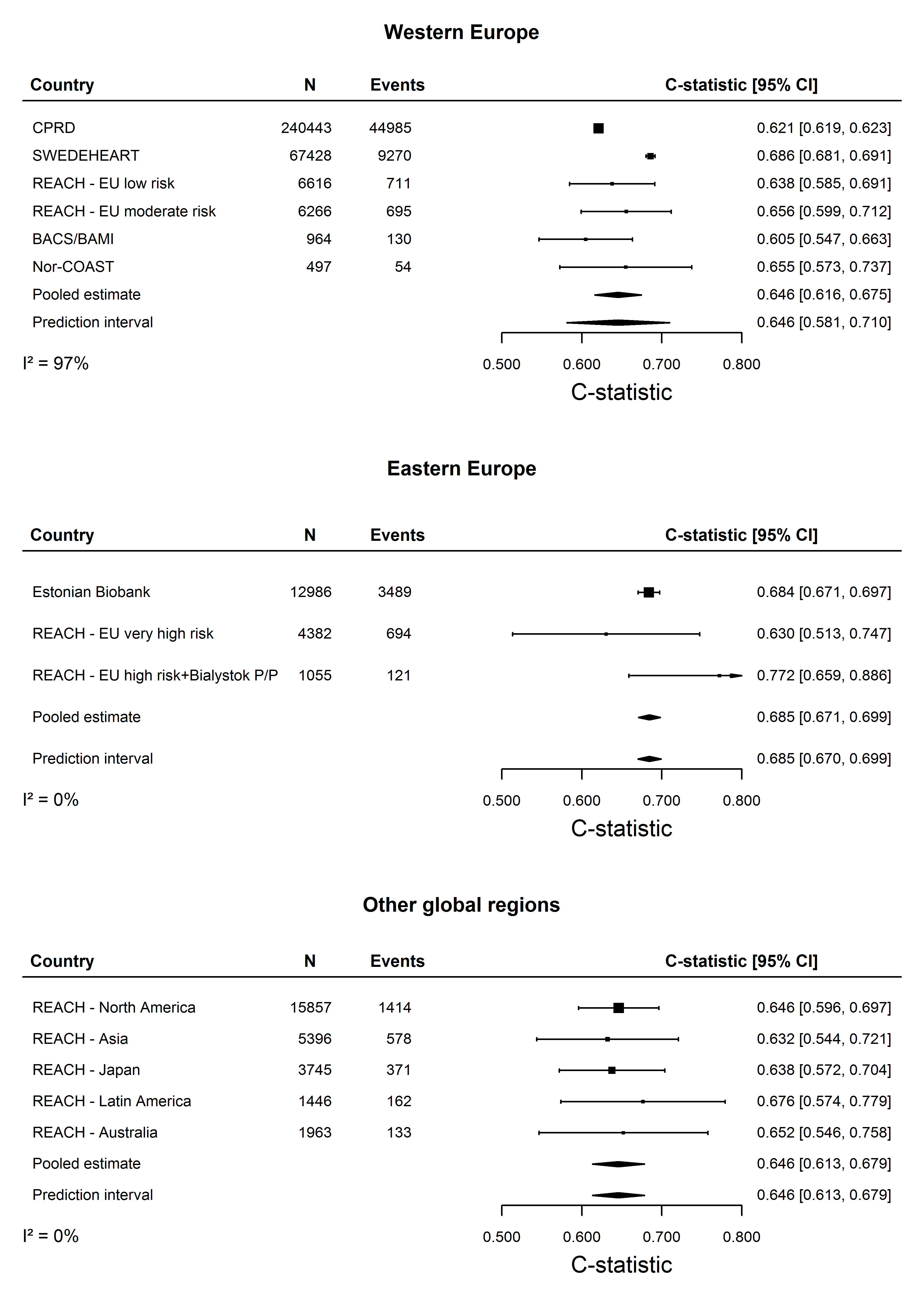


# **Supplementary Figure 3:** Calibration of the SMART2 risk score before recalibration in Western Europe


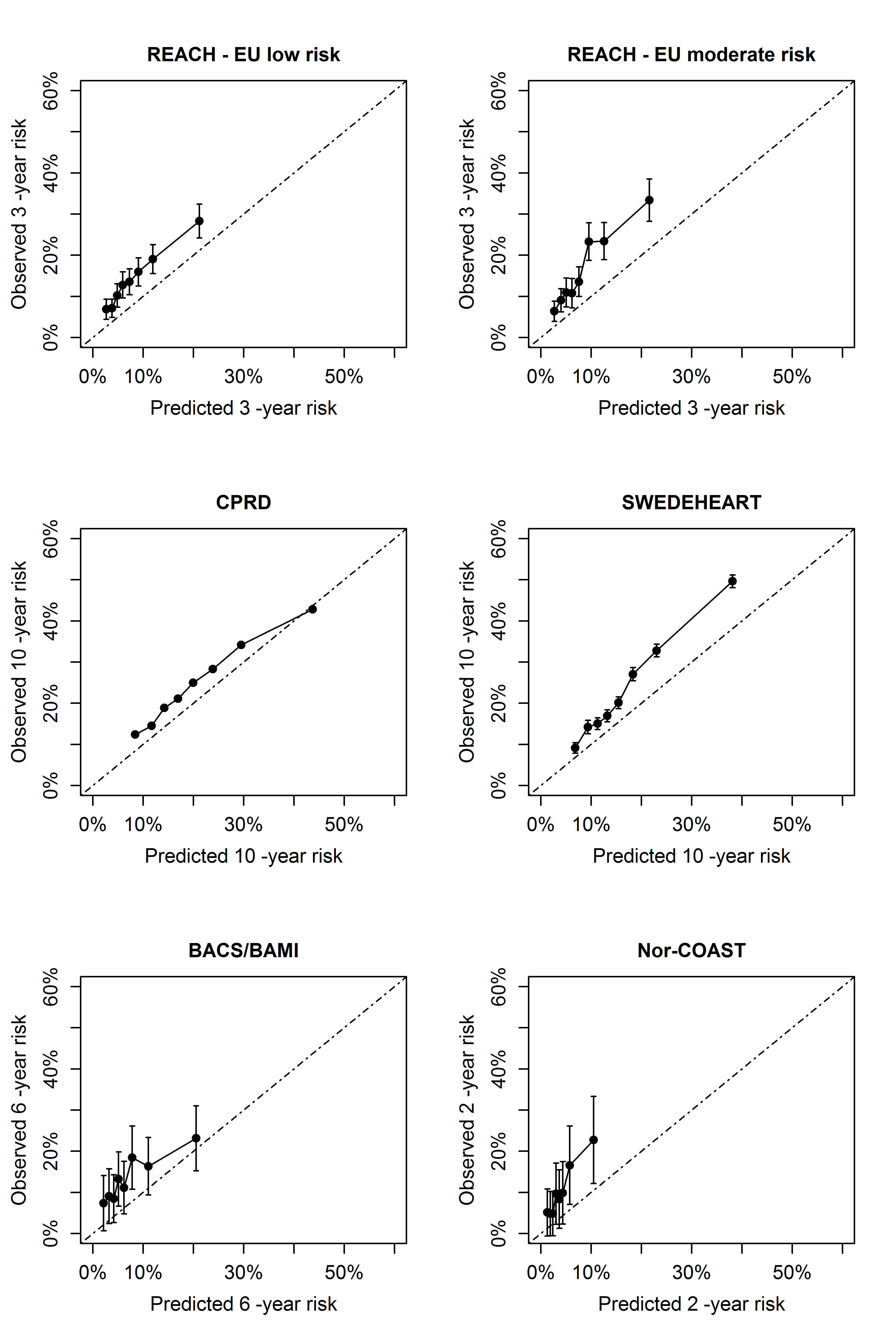


# **Supplementary Figure 4:** Calibration of the SMART2 risk score before recalibration in Eastern Europe


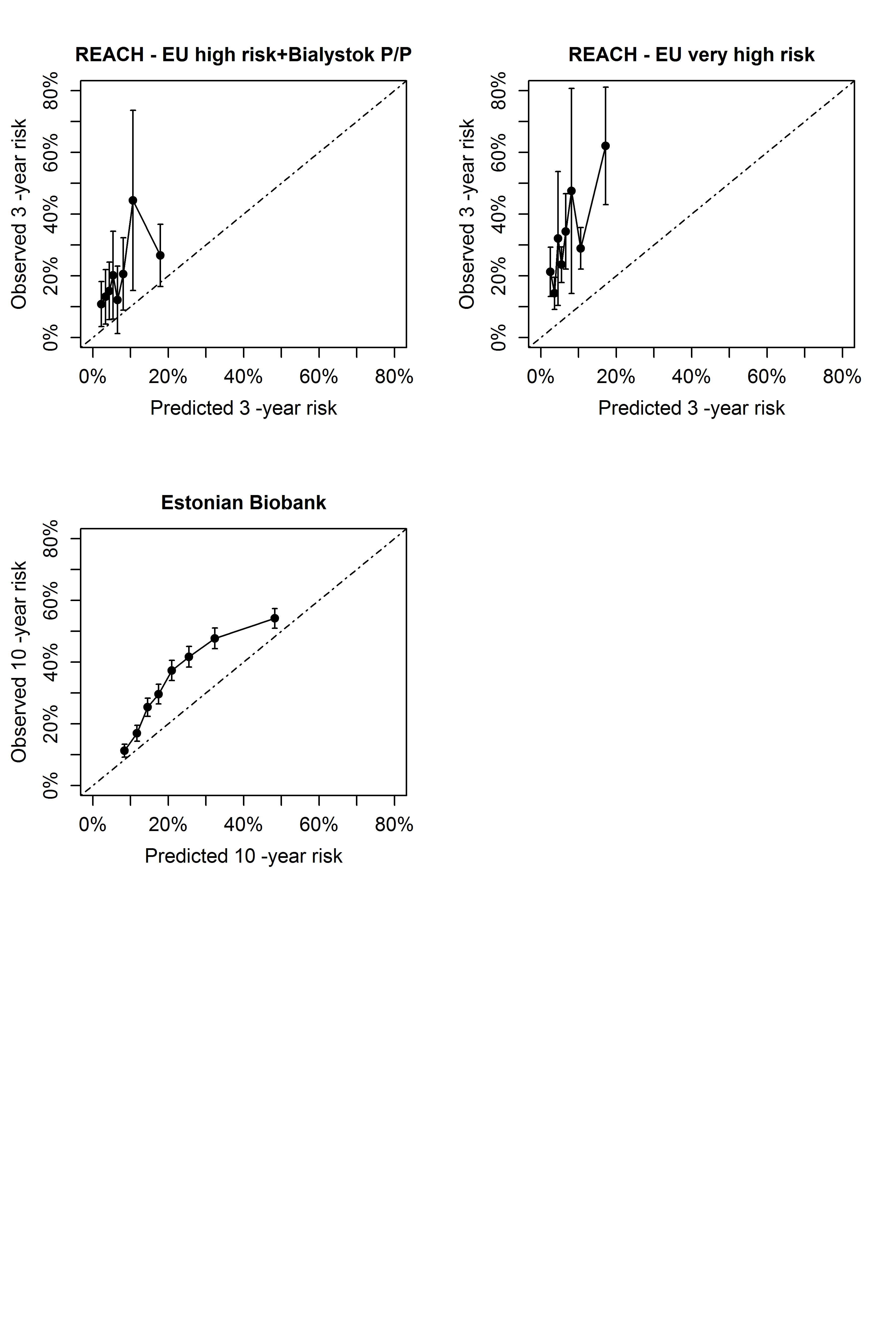


# **Supplementary Figure 5:** Calibration of the SMART2 risk score before recalibration in the Non-European external validation cohorts


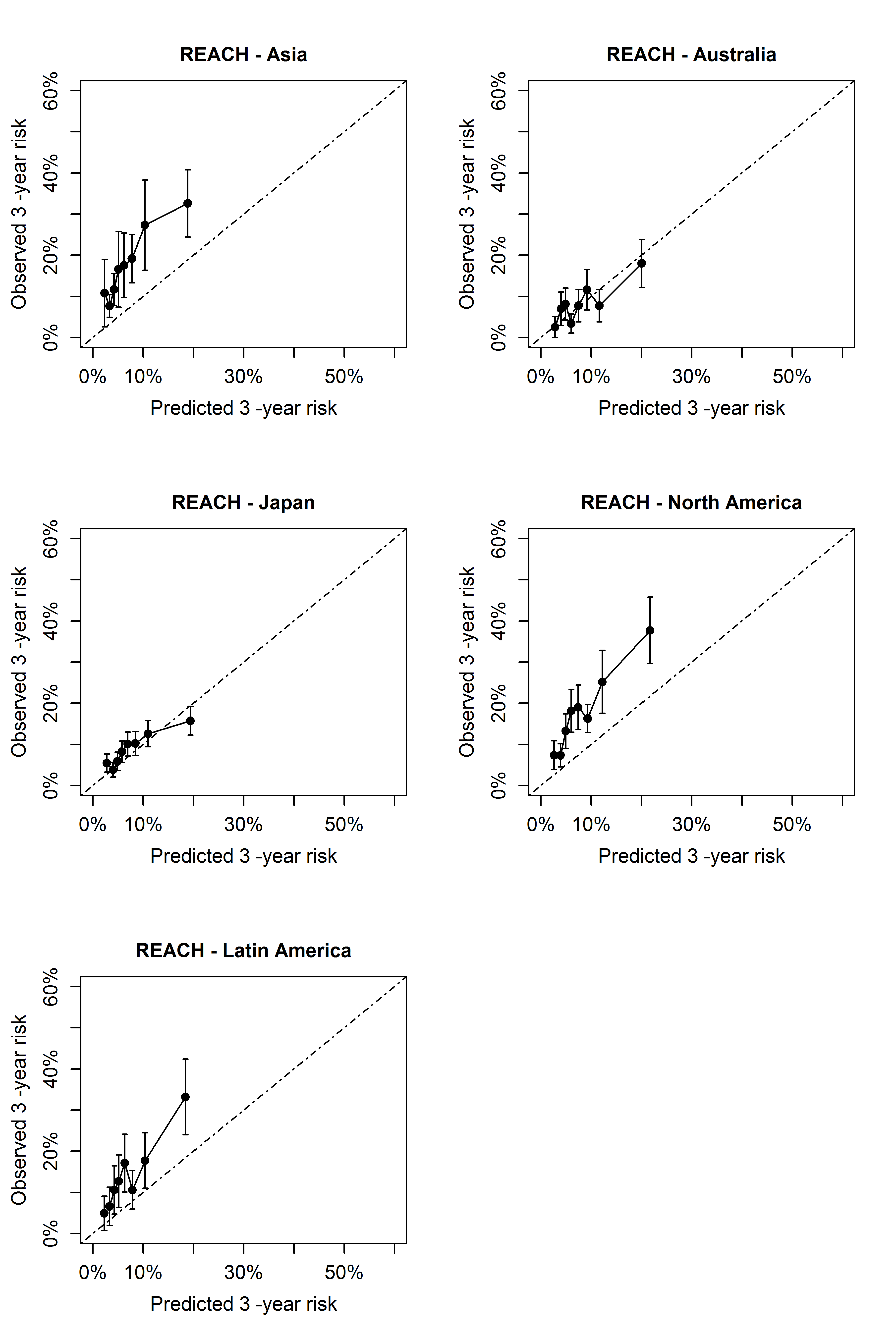


# **Supplementary Figure 6:** Net benefit of treatment intensification based on the SMART2 algorithm


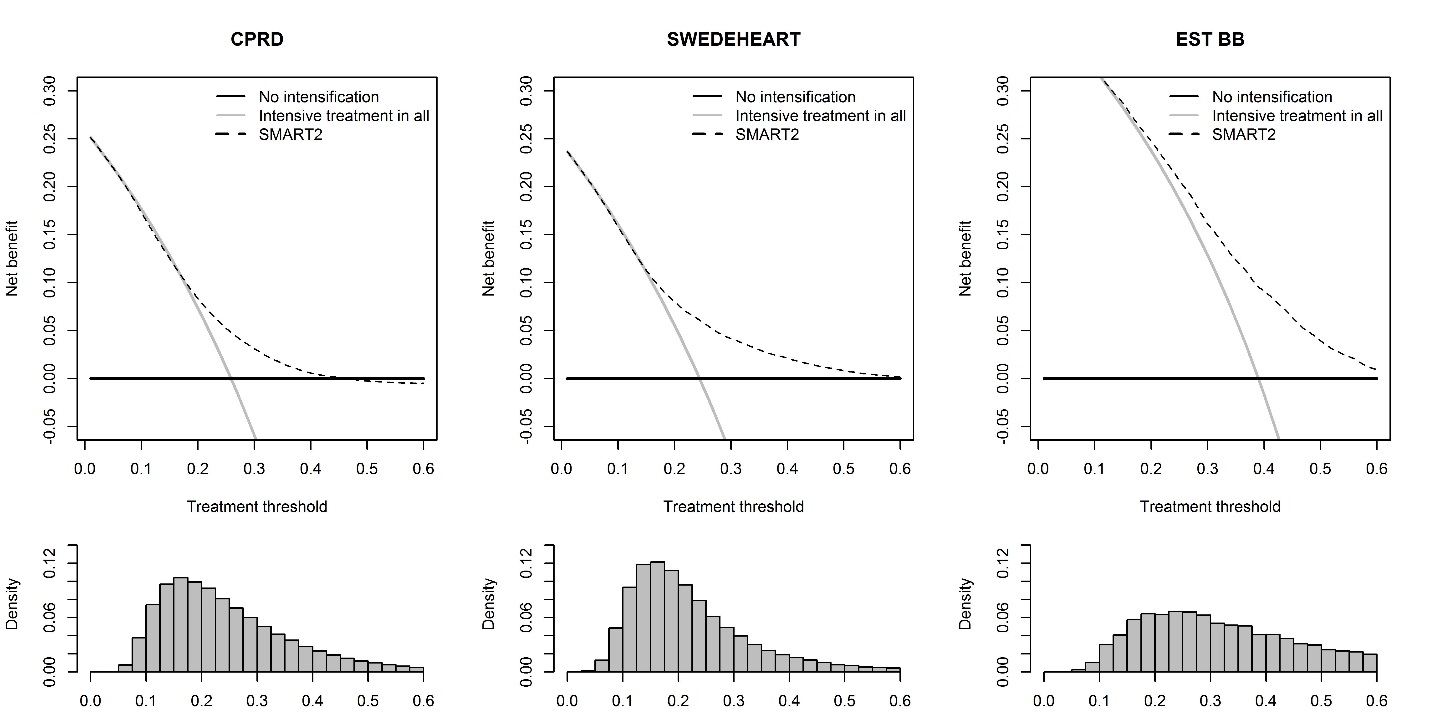


Results from the decision curve analyses in all cohorts with at least 10 years maximum follow-up. Each panel displays the net benefit of treatment intensification based on the SMART2 model (dashed line) against the treat all (gray line) and treat none (black line) approaches.

# **Supplementary Figure 7:** Illustration of the SMART2 risk score recalibrated to the different European regions


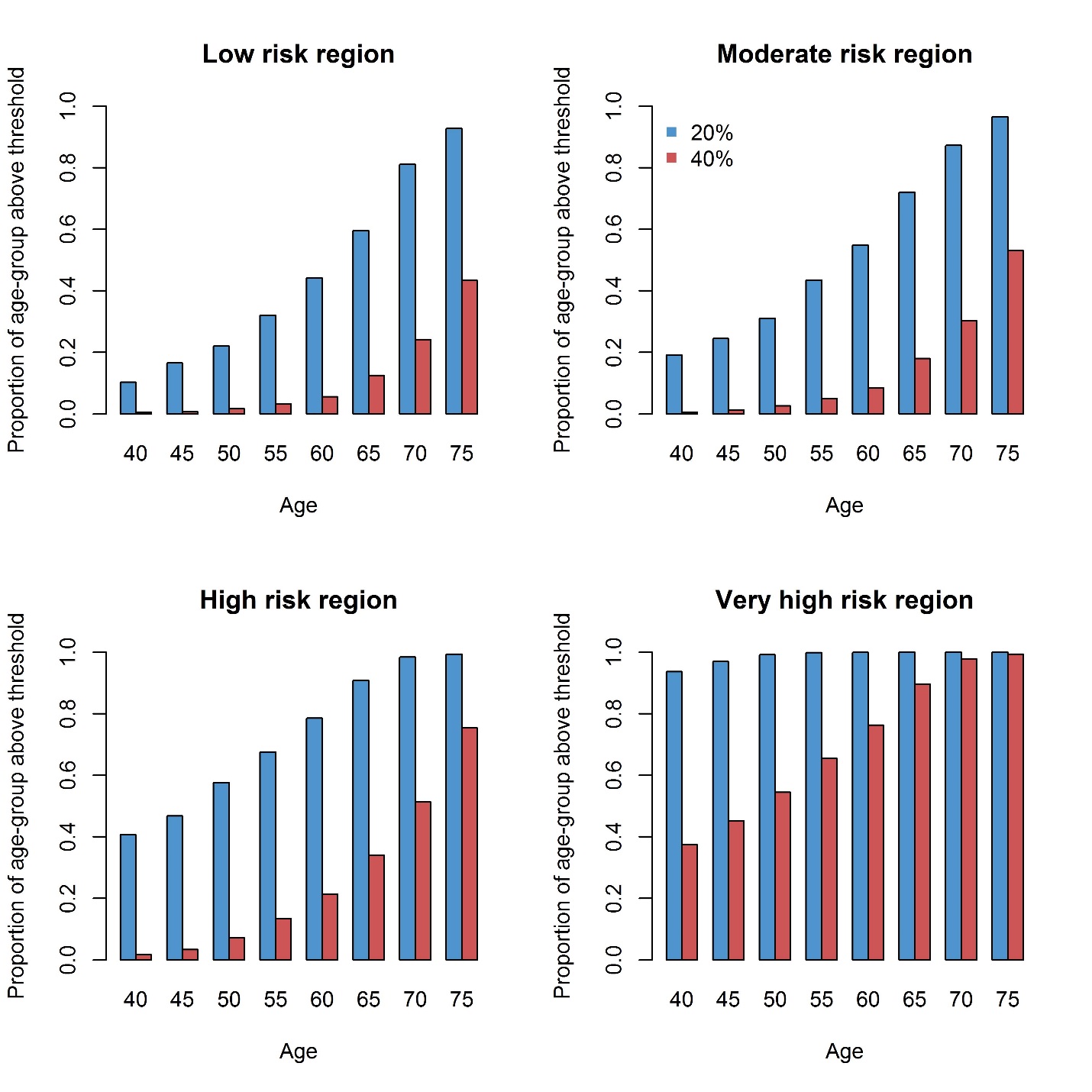


Expected proportion of individuals above the 20 and 40% 10 year risk thresholds in every risk region in Europe. Results were based on a simulation in UCC-SMART (n=8,355) assuming equal risk factor distributions across regions.

# **Supplementary Table 1:** Endpoint definitions

| **Fatal cardiovascular disease** | |
| --- | --- |
| Endpoints included | ICD10-codes |
| Hypertensive disease | I10-16 |
| Ischemic heart disease | I20-25 |
| Arrhythmias, heart failure | I46-52 |
| Cerebrovascular disease | I60-69 |
| Atherosclerosis/AAA | I70-73 |
| Sudden death and death within 24h of symptom onset | R96.0-96.1 |
|  |  |
| *Excluding the following* |  |
| Myocarditis, unspecified | I51.4 |
| Subarachnoid haemorrhage | I60 |
| Subdural haemorrhage | I62 |
| Cerebral aneurysm | I67.1 |
| Cerebral arteritis | I68.2 |
| Moyamoya | I67.5 |
|  |  |
| **Non-fatal events** |  |
| Non-fatal myocardial infarction | I21-I23 |
| Non-fatal stroke | I60-69 |
|  |  |
| *Excluding the following* |  |
| Subarachnoid hemorrhage | I60 |
| Subdural hemorrhage | I62 |
| Cerebral aneurysm | I67.1 |
| Cerebral arteritis | I68.2 |
| Moyamoya | I67.5 |

Endpoint definitions depend on cohort availability but where ideally defined as stated above

# **Supplementary Table 2:** Model parameters and prediction algorithm

| **Coeficients** |  | **Yearly baseline risks** | | **Expected-observed ratio** |  |
| --- | --- | --- | --- | --- | --- |
| **Predictor** | **Beta** | **Year of follow-up** | **Baseline risk** | **Risk region** | **Ratio** |
| Abdominal aortic aneurysm | 0.330356631 | 1 | 0.020867965 | Europe, low risk | 0.81590 |
| Age, years | -0.03496022 | 2 | 0.03481669 | Europe, moderate risk | 0.6973285 |
| Age squared, years | 0.000551072 | 3 | 0.047574783 | Europe, high risk | 0.5085825 |
| Aspirin or equivalent | -0.21072103 | 4 | 0.06257936 | Europe, very high risk | 0.2285371 |
| Coronary artery disease | 0.294701954 | 5 | 0.07887031 | Asia | 0.4043255 |
| eGFR (CKDEPI) | -0.03967521 | 6 | 0.094891922 | Australia | 1.0040808 |
| eGFR squared (CKDEPI) | 0.000218613 | 7 | 0.112217969 | Japan | 0.8825678 |
| hsCRP (log) | 0.151760173 | 8 | 0.129898521 | North America | 0.4714961 |
| Current smoking | 0.345583271 | 9 | 0.149001544 | Latin America | 0.5075670 |
| Cerebrovascular disease | 0.34831786 | 10 | 0.165822797 |  |  |
| Diabetes Mellitus | 0.318170659 |  |  |  |  |
| Non HDL-c (log) | 0.540364249 |  |  | **Mean linear predictor** | -0.0463729 |
| Peripherial artery disease | 0.22446658 |  |  |  |  |
| Systolic blood pressure | 0.018913154 |  | **Formula to calculate individual risks** | |  |
| Male sex | 0.287658743 |  |  |  |  |
| Years since first ASCVD diagnosis | 0.047699585 |  | ${CVDrisk}_{10}=1 -{{(1-baserisk}_{10})}^{exp({LP}_{i} -{LP}_{mean} -\log\left( {ratio}_{region} \right))}$ | ${LP}_{i}=\sum Coef *individual risk factors$ |  |
| Years since first ASCVD diagnosis, squared | -0.00164973 |  |  |  |  |

All model parameters required for individual predictions. For prediction on horizons shorter than 10 years, the yearly baseline risks of the respective years can be used in the formula. ASCVD = Atherosclerotic cardiovascular disease. hsCRP = High-sensitivity C-reactive protein, HDL = high density lipoprotein, eGFR = Estimated glomular filtration rate, LP = linear predictor, *_i_* = individual.

# **Supplementary Table 3:** Summary of recalibration procedure

| **Step 1: calculate crude SMART2 risk**  First, the non-recalibrated SMART2 10 year risk is predicted for all individuals in the data source. The mean of all these individual predicted risks is the ‘expected’ incidence.  ${CVDrisk}_{10}=1 -{{(1-baserisk}_{10})}^{\exp\left( {LP}_{i} -{LP}_{mean} \right)}$ |
| --- |
| **Step 2: calculate cumulative incidence**  In the data source, the cumulative incidence at 10 years is obtained, taking into account competing risks (R function *cuminc, cmprsk package*). This is the ‘observed’ incidence in the cohort. |
| **Step 3: Expected-observed ratio**  Above metrics are combined in the expected-observed ratio.  $EO{ratio}_{region}=\frac{mean\left( {CVDrisk}_{10} \right)}{{Cum.inc}_{10}}$  For the high risk region, there was no cohort as large and minimally selected as CPRD (low risk region) or SWEDEHEART (moderate risk region). Therefore, the expected-observed ratios from the individual cohorts were averaged to get a regional recalibration factor. |
| **Step 4: Calculate recalibrated SMART2 risks**  Individual recalibrated risks can be calculated by using the natural logarithm of the expected observed ratio.  ${CVDrisk}_{10}=1 -{{(1-baserisk}_{10})}^{\exp\left( {LP}_{i} -{LP}_{mean} -\ln\left( EO{ratio}_{region} \right) \right)}$ |

# **Supplementary Table 4:** Discriminative model performance with sex-specific or established ASCVD location-specific model derivation

| **REACH Western Europe (n=12,882)** | **C-statistic (95%CI)** | |
| --- | --- | --- |
|  | Overall derivation | Sex-specific derivation |
| **Total population** | 0.644 (0.629-0.659) | 0.642 (0.627-0.658) |
| **Men only** | 0.646 (0.629-0.662) | 0.645 (0.629-0.662) |
| **Women only** | 0.643 (0.623-0.662) | 0.639 (0.620-0.658) |
|  | Overall derivation | Location-specific derivation |
| **Total population** | 0.644 (0.629-0.659) | 0.645 (0.630-0.660) |
| **CAD patients only** | 0.663 (0.645-0.681) | 0.661 (0.643-0.679) |
| **CeVD patients only** | 0.650 (0.609-0.690) | 0.650 (0.609-0.690) |
| **PAD/AAA patients only** | 0.635 (0.606-0.664) | 0.634 (0.604-0.663) |

External model performance in terms of discrimination when repeating model derivation and validation separately for both sexes (top) and locations of established ASCVD (bottom) in comparison to using the whole dataset. CAD = coronary artery disease, CeVD = cerebrovascular disease, PAD = peripheral artery disease, AAA = abdominal aortic aneurysm.
